# Subtle Scientific Fallacies Undermine the Validity of Neuroendocrinological Research: Do Not Draw Premature Conclusions on the Role of Female Sex Hormones

**DOI:** 10.3389/fnbeh.2017.00003

**Published:** 2017-01-17

**Authors:** Michael P. Hengartner

**Affiliations:** Department of Applied Psychology, Zurich University of Applied Sciences (ZHAW)Zurich, Switzerland

**Keywords:** bias, science, research, hormones, depression, menopause, methodology, statistics

## Abstract

Major scientific flaws such as reporting and publication biases are well documented, even though acknowledgment of their importance appears to be lacking in various psychological and medical fields. Subtle and less obvious biases including selective reviews of the literature and empirically unsupported conclusions and recommendations have received even less attention. Using the literature on the association between transition to menopause, hormones and the onset of depression as a guiding example, I outline how such scientific fallacies undermine the validity of neuroendocrinological research. It is shown that in contrast to prominent claims, first, most prospective studies do not support the notion that the menopausal transition relates to increased risk for depression, second, that associations between hormone levels and depression are largely inconsistent and irreproducible, and, third, that the evidence for the efficacy of hormone therapy for the treatment of depression is very weak and at best inconclusive. I conclude that a direct and uniform association between female sex hormones and depression is clearly not supported by the literature and that more attention should be paid to the manifold scientific biases that undermine the validity of findings in psychological and medical research, with a specific focus on the behavioral neurosciences.

## Background

Based on extensive theoretical considerations, Ioannidis (2005a) drew the bold conclusion that, in biomedicine, “most published research findings are false”. Original studies subjecting psychological and medical research to close scrutiny have indeed provided ample evidence that many, and sometimes even a majority, of published findings are irreproducible, false positive, or severely inflated (Ioannidis, 2005b; Turner et al., 2008; Munafo et al., 2009; Bakker et al., 2012; Begley and Ellis, 2012; Open Science Collaboration, 2015; Müller et al., 2017); for reviews see (Nosek et al., 2012; Macleod et al., 2014; Naci and Ioannidis, 2015). More specifically, Open Science Collaboration (2015) tried to replicate 100 original studies published in 2008 in three leading psychology journals. While 97% of original studies had a significant result (i.e., *p* < 0.05), only 36% of the replication studies revealed a significant result, even though they all had high statistical power. Moreover, in the original studies the average effect size was *r* = 0.403 (SD = 0.188), whereas in the replication studies it was only *r* = 0.197 (SD = 0.257), suggesting that the evidence for the original findings is rather weak (Open Science Collaboration, 2015). Very recently, Müller et al. (2017) meta-analyzed neuroimaging studies in persons with unipolar depression conducted between 1997 and 2015 and found not a single functional neuro-pattern that replicated consistently across studies, suggesting that the grand majority of published findings are irreproducible or false-positives. Finally, Contopoulos-Ioannidis et al. (2003) identified a total of 101 articles published between 1979 and 1983 in six leading biomedical journals, which clearly stated that the technology studied had novel therapeutic or preventive promises. However, most findings turned out to be false-positives or gross overestimations; by October 2002, only 27 of the promising technologies had resulted in at least one published randomized trial, five articles resulted in interventions with clinical licence, but only one finding lead to the development of an intervention that has been used extensively for the licensed indications (Contopoulos-Ioannidis et al., 2003).

In psychology/psychiatry the rate of positive findings (i.e., *p* < 0.05) is currently around 92% (Fanelli, 2010). Because 92% of all published results cannot possibly be true-positives given the average sample size and statistical power in published research, obviously strong and systematic biases are taking effect (Bakker et al., 2012; Button et al., 2013). There are various reasons for this excess significance bias, including foremost, reporting and publication biases (Ferguson and Heene, 2012; Glasziou et al., 2014; Ioannidis et al., 2014b) as well as methodological biases such as statistical misconduct and questionable research practices (Simmons et al., 2011; John et al., 2012; Ioannidis et al., 2014a). Because many conflicting results and negative findings are not objectively reported or published, the scientific literature is systematically biased towards spectacular positive findings (Young et al., 2008; Nosek et al., 2012), which leads to inflated meta-analytic estimates of effect sizes (Cuijpers et al., 2010; Ioannidis, 2011; Bakker et al., 2012). As a result, effectiveness of pharmaceutical drugs and impact of neurobiological and psychological findings are systematically overestimated (Ferguson and Heene, 2012; Button et al., 2013; Turner, 2013).

However, there are also subtle and less obvious fallacies. Sometimes conflicting results such as negative findings are sufficiently published, but these are simply not adequately acknowledged and considered (Jannot et al., 2013; Chalmers et al., 2014). That is, many researchers selectively review the literature and come to rather arbitrary conclusions that are not unequivocally supported by the published evidence (Ioannidis et al., 2007; Tatsioni et al., 2007; Saraga and Stiefel, 2011). Using the literature on the association between transition to menopause, hormonal changes and the onset of depression as a guiding example, I will thoroughly outline that many prominent claims do not hold up to close scrutiny and are therefore grossly exaggerated or false. I will ponder my arguments by testing two prevalent claims, the first being that the transition to menopause increases the risk for depression and the second that changing hormone levels directly and uniformly increase the risk for depression.

## First Claim: The Transition to Menopause Increases the Risk for Depression

That claim has been put forward by various authors of original studies (e.g., Bromberger et al., 2011; Joffe et al., 2016) and narrative reviews (e.g., Freeman, 2010; Soares, 2010). For instance, Bromberger et al. (2011) stressed that “we have moved from the “belief” that women were particularly susceptible to depression after the menopausal transition to the current empirically supported conclusion that middle-aged women are at a greater risk for depression during the transition than before [references]” (p. 1879). In addition, Soares (2010) stated that “Unlike cross-sectional studies, most prospective studies [references] have systematically confirmed the menopausal transition as a period of heightened risk for development of depressive symptoms and/or depression” (p. 2). Both strengthen their statement by citing various prospective observational studies that ostensibly confirmed the association between depression and transition to menopause. However, that view does not withstand close scrutiny and has been challenged by various contradictory findings (Judd et al., 2012; Rössler et al., 2016). First of all, that claim misses that most prospective studies did actually NOT report an association (Vesco et al., 2007; Rössler et al., 2016), so here many researchers are producing a severe confirmation bias by selectively reviewing a minority of studies with positive results that are not representative of the broader literature (Ioannidis et al., 2007; Tatsioni et al., 2007; Chalmers et al., 2014). Of concern is also the substantial content overlap between depression and menopause symptoms such as sleep disturbance, fatigue and irritability (Judd et al., 2012; Davis et al., 2015). Part of the covariance between menopause and depression symptoms is therefore tautological, which artificially inflates the strength of association. This could at least in part explain why some studies relying of self-report inventories of depression find a significant association anyway. In addition, various prospective studies that did report a positive association appear to be systematically biased due to the inadequate statistical procedure of dichotomization of a continuous measure of depression. As demonstrated by Rössler et al. (2016), when a continuous measure of depression is artificially dichotomized into categories of present vs. absent based on arbitrary cut-offs, false-positive associations with menopause stages can emerge. That the dichotomization of continuous variables conveys severe bias is widely acknowledged in the methodological literature (Ragland, 1992; MacCallum et al., 2002; Royston et al., 2006). Unfortunately, that ill-advised practice is still very prevalent in psychological and medical research. Rössler et al. (2016) name further biases in highly cited reports of positive associations between menopause transition and the onset of depression. These comprise among others overfitting and multiple adjustments in multivariable regression models, which can also produce inflated or false-positive associations (Babyak, 2004; Simmons et al., 2011).

In conclusion, firstly, most prospective studies failed to provide support for a positive association between the transition to menopause and the occurrence of depression (Vesco et al., 2007; Rössler et al., 2016). Unfortunately, when looking at citation patterns one will easily recognize that the studies with positive findings receive much more attention than studies with negative findings (Kjaergard and Gluud, 2002; Jannot et al., 2013; Glasziou et al., 2014). For example, the negative findings from Avis et al. (1994) and Kaufert et al. (1992) received 499 and 380 citations (as of October 2016), whereas the much more recent positive findings by Cohen et al. (2006) and Freeman et al. (2006) received already 562 and 538 citations. Second, some studies that did report a positive association applied problematic statistical procedures indicating that data were possibly “tortured until they confess” (Wagenmakers et al., 2012) to obtain statistically significant associations at *p* < 0.05 (Simmons et al., 2011; John et al., 2012; Ioannidis et al., 2014a). That phenomenon is also well documented as p-hacking and referred to journals’ and researcher’s aversion towards negative findings (Young et al., 2008; Ferguson and Heene, 2012; Nosek et al., 2012).

## Second Claim: Hormone Levels Directly Influence Depressive Symptoms

Due to a selective reference to positive associations between the transition to menopause and depression (see above), it was concluded that the hormonal changes during that period may cause depression. As a result, hormone replacement therapy has been proposed as a potent first-line treatment for depression in peri-menopausal women (Riecher-Rössler and de Geyter, 2007; Georgakis et al., 2016). As above, that claim is not sufficiently supported by the literature. First of all, the association between hormone levels and depression during the transition to menopause is unclear, that is, positive associations are merely anecdotal and have not been replicated thus far (see reviews by Freeman, 2010; Vivian-Taylor and Hickey, 2014). So, once rapidly increasing FSH relates to lower risk of depression (Freeman et al., 2004) and once to higher risk of depression (Ryan et al., 2009), then again it is testosterone (Bromberger et al., 2010) and according to another study it is the fluctuating estradiol (Freeman et al., 2006) that causes depression. Moreover, as we would expect from a true null association, it comes as no surprise that some studies did not find any association between hormone levels and depression at all (Woods et al., 2008; Bromberger et al., 2011). In accordance, results from randomized clinical trials testing the efficacy of hormone therapy for depressive symptoms in menopausal women are also inconsistent (Soares et al., 2001; Morrison et al., 2004; Joffe et al., 2011) and provide overall only weak evidence in support of hormone therapy for depressed peri-menopausal but not post-menopausal women (see systematic review by Rubinow et al., 2015). However, due to substantial reporting and publication biases in the evaluation of drug trials (Turner, 2013; Naci and Ioannidis, 2015), these suggestions must be taken with reservation. Also, in initially non-depressed women who make the transition to menopause, hormone therapy has no preventive value (Rubinow et al., 2015). Recently, Georgakis et al. (2016) showed in a comprehensive meta-analysis that in women who naturally enter menopause (*n* = 67434), higher age at menopause was associated with a marginally smaller risk of depression (OR = 0.98 for a 2-year increment, *p* < 0.05). They supposed that this association was due to estrogen exposure and concluded that estrogen-based therapies could be useful to prevent depression in women who naturally enter the menopause before population average, even though the systematic review by Rubinow et al. (2015) concluded that hormone therapy has no preventive value. In a commentary on Georgakis et al. (2016), Hengartner (2016) transformed that odds ratio effect size into the number needed to treat (NNT), which is a convenient measure to quantify the effectiveness of medical treatments (Cook and Sackett, 1995). He showed that the NNT was 500, suggesting that 500 women would need to undergo continuous estrogen substitution for 2 years in order to prevent depression in only one woman (Hengartner, 2016). Of course, such a treatment is ineffective and no option to prevent depression in women making the transition to menopause. Unfortunately, misleading and empirically unsupported conclusions are quite common in medical research (Ioannidis et al., 2007; Saraga and Stiefel, 2011). In addition, another very prevalent scientific fallacy in contemporary psychological and medical research is the confusion of statistical significance with practical significance (Kirk, 1996). When the sample size is large enough (say *n* > 10000), even negligibly small deviations from the null that bear absolutely no practical implications will become statistically significant (Cohen, 1994; Hengartner, 2016).

In conclusion, a critical examination of the literature does not support the view that sex hormone levels uniformly relate to depression. A systematic review of the literature indicates that most published findings are anecdotal, irreproducible and inconsistent, especially in the behavioral neurosciences (Ioannidis, 2011; Rosmalen and Oldehinkel, 2011; Ioannidis et al., 2014b; Sundström Poromaa and Gingnell, 2014; Müller et al., 2017). Nevertheless, many researchers tend to selectively review the literature (Tatsioni et al., 2007; Jannot et al., 2013) and to uncritically draw conclusions that are not sufficiently supported by the literature (Ioannidis et al., 2007; Saraga and Stiefel, 2011; Chalmers et al., 2014). Premature treatment recommendations can expose thousands of patients to treatments that are largely ineffective or that may cause more harm than good (Naci and Ioannidis, 2015). In this respect it is also necessary to evaluate the net benefit of a given therapy. Too often researchers equate statistical significance with clinical significance (Hengartner, 2016), ignoring that statistical significance does not allow to make inferences on the effectiveness of a given treatment (Cohen, 1994; Kirk, 1996).

## Concluding Remarks

By now it is increasingly documented that some psychological and medical science is systematically flawed (Ioannidis, 2005a; Ferguson and Heene, 2012; Pashler and Harris, 2012; Macleod et al., 2014). However, both researchers and journals have an aversion towards null results, because they neither boost a researcher’s career nor a journal’s reputation (Young et al., 2008; Nosek et al., 2012). Major scientific fallacies such as reporting and publication biases cause a systematic overestimation of reported effect sizes to the point that many associations in the scientific literature are eventually marginally small or false-positives (Bakker et al., 2012; Ferguson and Heene, 2012; Ioannidis et al., 2014b; Naci and Ioannidis, 2015). However, reporting and publication biases are only the tip of the iceberg. Using the association between transition to menopause, hormones and depression as a guiding example, I demonstrated that, first, the scientific literature is often selectively reviewed and synthesized (Tatsioni et al., 2007) and second, that unsupported conclusions and treatment recommendations are readily made (Saraga and Stiefel, 2011). Specifically, in contrast to prominent claims that an increased risk for depression during the transition to menopause is clearly confirmed by the literature (Soares, 2010; Bromberger et al., 2011), a critical examination of the literature reveals that most prospective studies did not show a meaningful association (Vesco et al., 2007; Rössler et al., 2016). As the transition to menopause is a critical life event accompanied by major psychosocial changes, the most promising explanation is that these psychosocial risk factors may predispose vulnerable women to depression, but not the hormonal changes *per se* (Kaufert et al., 1992; Nelson, 2008; Rössler et al., 2016). In line with that notion it has been shown that an association between hormonal changes during the menopausal transition and depression is largely inconsistent (compare: Freeman et al., 2004; Woods et al., 2008; Ryan et al., 2009; Bromberger et al., 2010) and that the efficacy of hormone therapy to treat depression is at best inconclusive (Nelson, 2008; Rubinow et al., 2015). Therefore, recommending estrogen as a useful (preventive) treatment for depression in peri-menopausal women appears misguided and premature.

I strongly believe that hormones are crucial for affect and behavior. However, their effect on psychopathology is certainly not direct and uniform, otherwise research would have shown it. It is therefore supposed that hormones modulate complex psychobiological mechanisms such as the stress response system, and that these, further modulated through even more psychobiological factors, impact on psychopathology (Zahn-Waxler et al., 2008; Naninck et al., 2011; Handa and Weiser, 2014). However, research into complex neurobiological systems is just at its beginning. As long as the neurosciences are so fundamentally hampered by manifold methodological biases, including notoriously underpowered samples, opaque experimental designs, almost unrestricted flexibility in data analysis and flawed statistical methods (e.g., Kriegeskorte et al., 2009; Carp, 2012; Button et al., 2013; Eklund et al., 2016), increased consistency and reproducibility of neurobiological research findings will not be readily achieved. A particularly striking example was provided by the senior authors of the Tracking Adolescents’ Individual Lives Survey (TRAILS; Rosmalen and Oldehinkel, 2011). This large multisite project was designed to focus on the effects of cortisol on psychopathology. Rosmalen and Oldehinkel (2011) self-critically admit, that when they soon realized that the initial cross-sectional analyses yielded mainly null results or negligibly weak associations, the principal investigators started to test manipulations and different definitions of predictor and outcome variables; they arbitrarily included varying sets of potential confounders, and they used alternative statistical procedures in order to obtain statistically significant associations. The result of these inappropriate data manipulations (see Simmons et al., 2011) is that they achieved to publish spectacular positive findings in the leading journals. However, ultimately these confusing findings were highly inconsistent and most likely irreproducible false-positives that did not advance our knowledge in this field (Rosmalen and Oldehinkel, 2011). Looking at other hot topics in the behavioral neurosciences (e.g., Sundström Poromaa and Gingnell, 2014), one will easily recognize that this issue is the rule rather than the exception. It will therefore presumably take some time until we know how sex hormones and complex neurobiological systems influence psychopathology. In the meantime we are best advised to abstain from oversimplified and premature conclusions and instead to pay more attention to scientific and methodological biases.

## Author Contributions

MPH wrote the entire manuscript.

## Conflict of Interest Statement

The author declares that the research was conducted in the absence of any commercial or financial relationships that could be construed as a potential conflict of interest.
